# Dietary habits, depression and obesity: an intricate relationship to explore in pediatric preventive strategies

**DOI:** 10.3389/fped.2024.1368283

**Published:** 2024-03-08

**Authors:** Valeria Calcaterra, Virginia Rossi, Vittoria Carlotta Magenes, Paola Baldassarre, Roberta Grazi, Martina Loiodice, Valentina Fabiano, Gianvincenzo Zuccotti

**Affiliations:** ^1^ Pediatric and Adolescent Unit, Department of Internal Medicine, University of Pavia, Pavia, Italy; ^2^ Pediatric Department, “Vittore Buzzi” Children’s Hospital, Milan, Italy; ^3^ Department of Biomedical and Clinical Science, Università Degli Studi di Milano, Milan, Italy

**Keywords:** dietary habits, diet, depression, obesity, children, adolescents, prevention, mental health

## Abstract

Obesity and depression represent major health problems due to their high prevalence and morbidity rates. Numerous evidences elucidated the connections between dietary habits and the incidence or severity of depression. This overview aims to investigate the intricate relationship between dietary patterns and depression with the objective of elaborating preventive strategies for childhood obesity. Literature data recognized that there is a link between mood and food choices, with certain foods selected for their impact on the brain's reward centers. This behavior parallels the one observed in substance addiction, suggesting a specific neural mechanism for food addiction that contributes to overeating and obesity. It is important to note the significant correlation between obesity and depression, indicating a shared biological pathway influencing these conditions. Stress substantially affects also eating behaviors, often leading to increased consumption of pleasurable and rewarding foods. This can trigger a cycle of overeating, weight gain, and psychological distress, exacerbating mood disorders and obesity. In addition, consumption of certain types of foods, especially “comfort foods” high in fat and calories, may provide temporary relief from symptoms of depression, but can lead to long-term obesity and further mental health problems. Understanding these complex interactions is critical to developing preventive strategies focusing on dietary, emotional, and environmental factors, thereby reducing the risk of obesity and mood disorders.

## 1 Introduction

Obesity and depression represent major health problems due to their high prevalence and morbidity rates, contributors to disease burden also within the pediatric population. Adolescence, marked by significant psychosocial and physical changes, heightens the likelihood of co-occurring obesity and depression (1, 2). While depression is a common mental health disorder, its multifaceted etiological mechanisms remain not completely understood. Typical symptoms include a persistent low mood, reduced interest in usual activities, a generally negative outlook that hinders daily life, and pessimistic expectations about the future. The spectrum of effects and severity varies, ranging from mild cases characterized by a loss of interest and anhedonia to severe instances involving self-harm and suicide (1, 2).

**Figure 1:**
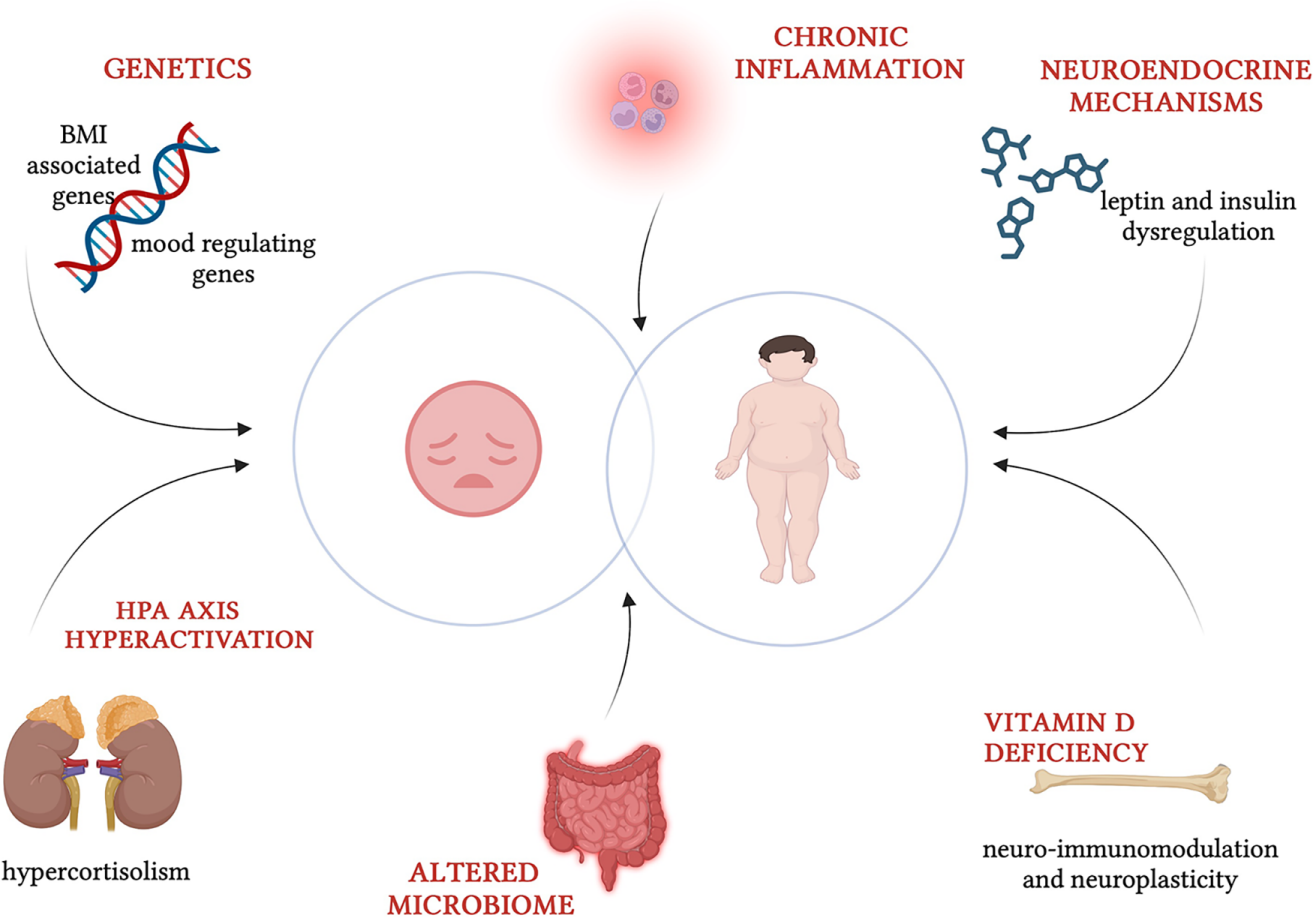
Common pathogenic factors in childhood obesity and depression (created with BioRender.com).

The frequent co-occurrence of obesity and depression indicates a combined effect of various factors (Figure 1). The relationship is complex and bidirectional, but not yet fully explored. A variety of non-modifiable factors, like genetics, and modifiable factors, including psychological and environmental aspects (diet, lifestyle and social support), contribute to this intricate interplay (3). Obesity, characterized by hormonal imbalances and chronic inflammation, may play a role in precipitating depressive symptoms. Indeed, children and adolescents who are overweight or obese may often experience negative body image, stigmatization and low self-esteem increasing their vulnerability to depression. Additionally, depression may affect hormones involved in appetite-satiety circuit regulation. Sedentary lifestyle and emotional eating, intertwined with unhealthy dietary habits, represent shared aspects of both conditions (4). Several studies also highlight numerous common biological mechanisms, that are likely involved in causing both obesity and depression. These include inflammation, dysregulation of the hypothalamic-pituitary-adrenocortical axis, poor glycemic control, and impairment in neurotransmitter and neuroendocrine systems through melanocortinergic leptin-brain-derived neurotrophic factor (BNDF) signaling (5). The comprehensive exploration of these shared mechanisms and contributing factors is essential for a deeper understanding of the intricate interplay between obesity and depression, paving the way for more targeted interventions and therapeutic strategies.

Over the past few decades, numerous systematic reviews and meta-analyses have established evidence elucidating the connections between dietary patterns, food quality and categories, macronutrients and micronutrients, and the incidence or severity of depression. Established findings highlight a negative relationship between the occurrence of depression and the intake of Mediterranean and traditional diets characterized by high levels of complex carbohydrates, B vitamins, omega-3 fatty acids and certain amino acids. Diets rich in fruits, vegetables, fish and meat have been associated with reduced symptoms of depression. Conversely, diets rich in processed or junk foods, saturated fats and sugary drinks have been linked to an increased risk of depression in adolescents (1, 3).

This overview aims to investigate the intricate association between dietary patterns and depression with the objective of elaborating preventive strategies for obesity in child and adolescent and investigating plausible impact of dietary interventions on managing depression.

## 2 Methods

We conducted a narrative review (6, 7) to examine the relationship between dietary patterns and depression in children and adolescents with obesity. We performed a comprehensive search of literature on the PubMed database and Cochrane Library, considering English language publications from the last 25 years. Our review encompassed a diverse range of publications: original research papers, systematic reviews, meta-analyses and longitudinal studies. These encompassed studies involving both adult and pediatric populations, with a specific focus on the pediatric age group; specifically, all pediatric articles were included, whereas concerning adult data, the authors incorporated the most significant findings from adult studies into the review. The keywords used in our search and the count of articles identified and scrutinized for each section are detailed in Table 1. We started with an initial pool of 230 articles, then narrowed it down by screening abstracts (*n* = 190) and subsequently conducting an in-depth review of the full texts of pertinent papers (*n* = 89).

**Table 1:** List of keywords and number of articles found and analyzed for each paragraph.

Paragraph	Keywords	Total of articles found	Relevant articles (Fully read)
Depression and pediatric obesity	“pediatric obesity” OR “overweight” AND “depression” OR “mental health” AND “children”	55	45 (8)
Interaction between mood, emotional state, and feeding behaviors	“mood” OR “depression” AND “emotional state” AND “feeding behavior” OR “dietary patterns” OR “dietary habits” OR “emotional eating” OR “nutrition”	65	50 (9)
Dietary habits and the risk of depression	“mood” OR “depression” AND “feeding behavior” OR “dietary patterns” OR “dietary habits”	60	55 (10)
Benefit of Diet Interventions on Obesity and Depression	“depression” OR “mood” AND “food” OR “nutrition” AND	50	40 (10)
	“obesity” OR “eating disorders” OR “nutritional psychiatry”		

These articles were meticulously analyzed to facilitate an informed and critical discussion. Furthermore, we checked the references of all reviewed articles.

**Figure 2:**
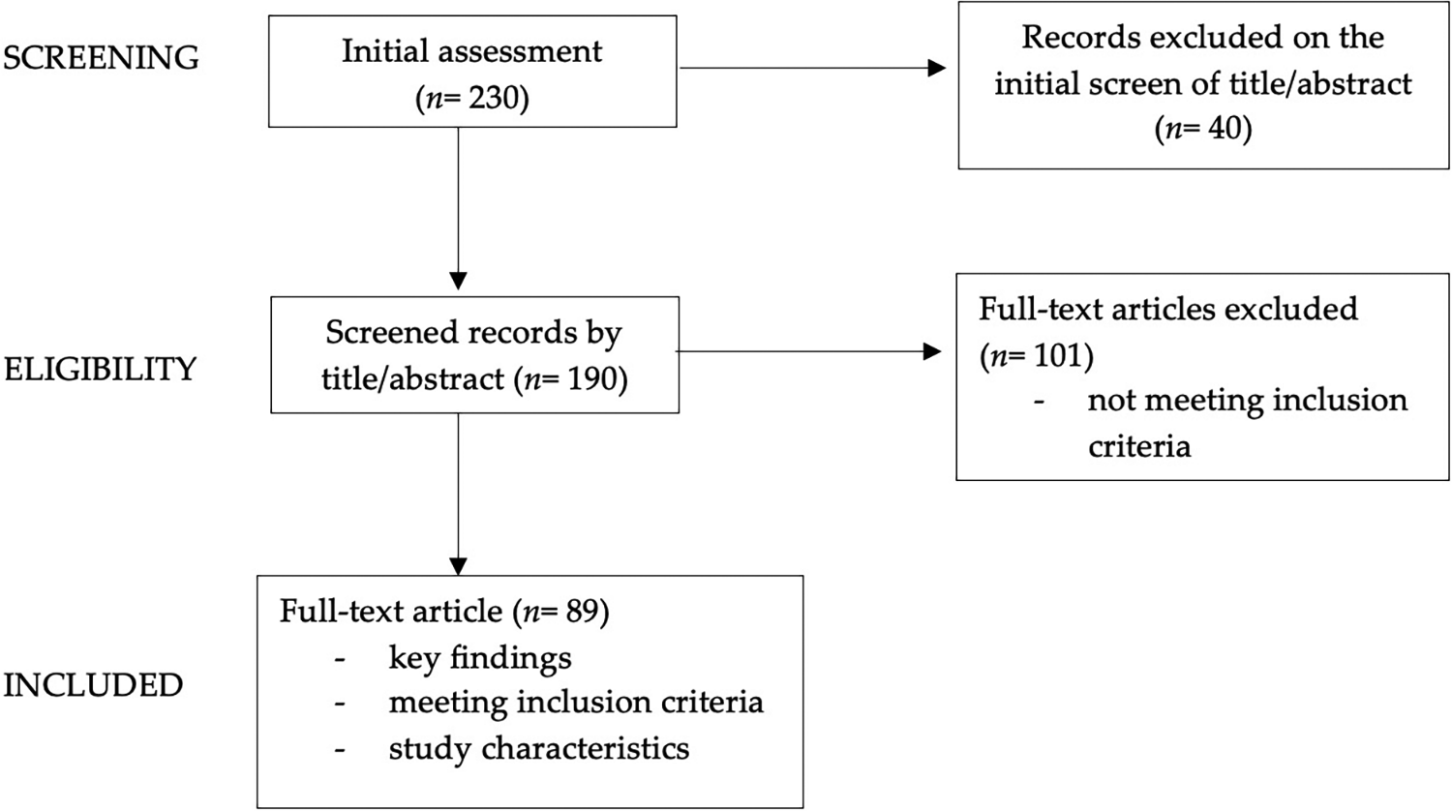
Flow chart showing the process of paper selection and exclusion.

In Figure 2, the diagram showing graphically the process of papers selection and exclusion is reported.

## 3 Depression and pediatric obesity

### 3.1 Relationship between depression and obesity

Childhood obesity poses a pervasive global public health challenge, with far-reaching consequences and associated comorbidities that jeopardize the well-being of forthcoming generations (11). While the undeniable physical ramifications of childhood obesity demand swift intervention, it is equally imperative to acknowledge the significant impact of excess weight on mental health and quality of life.

Depression impacts over 320 million individuals globally, recognized by the World Health Organization (WHO) as an emerging global health issue and a leading contributor to the worldwide disease burden (12–14). WHO estimates that mental health conditions are responsible for more than 15% of the burden of disease and injury among teens. Depression is the fourth leading cause of illness, and 20% of teenagers manifest some level of depressive symptoms or anxiety (2). This underscores the critical imperative for timely interventions aimed at reducing its prevalence and averting detrimental long-term social consequences. This condition manifests through persistent feelings of sadness, hopelessness, disinterest, low self-esteem, and frequent thoughts of death. Moreover, it can profoundly impair one's ability to handle everyday challenges, including work, ultimately eroding the individual's life skills, leading to disability, and even premature death (12–15).

Although weight loss in the absence of intentional dieting and reduced appetite are more commonly observed symptoms of depressive disorder, the Diagnostic and Statistical Manual of Mental Disorders, fifth edition (DSM-5) diagnostic criteria for a major depressive episode encompass weight gain and an increase in appetite (16). The correlation between fluctuations in weight and depression, whether occurring concurrently or at distinct times, suggests a potential shared etiology or a causal relationship where one may precipitate the other.

Sutaria et al. (17) conducted the most Comprehensive meta-analysis to date, incorporating twenty-two studies with prospective, retrospective, or cross-sectional designs. Their analysis encompassed data from 143,603 children aged 18 years or younger, drawn from general population or community settings. A noteworthy finding emerged regarding obese female children, who exhibited 44% increased odds of depression compared to their normal-weight counterparts, while no significant association was identified between overweight status in children and depression, nor among specific subgroups of obese or overweight males and depression.

Comparable findings were yielded by Moradi et al. (4), who conducted a meta-analysis comprising 28 observational studies. Their investigation not only examined the potential link between weight excess and obesity but also explored the correlation between body mass index (BMI) and the prevalence of additional psychological conditions, including low self-esteem and body dissatisfaction (4). Indeed, existing literature underscores the critical role of body dissatisfaction, marked by anxieties related to weight, body shape, and body fat. This phenomenon is of utmost importance, as it substantially contributes to the emergence of low self-esteem, thereby predisposing individuals in this age group to adverse mental health outcomes, notably depression (18).

Zhao et al. (19) conducted a cross-sectional study focusing on adults with overweight and obesity in the U.S., finding that a larger waist circumference or abdominal obesity was significantly linked to a higher probability of experiencing depressive symptoms. Hamer et al. (20) carried out a longitudinal study on adults with obesity, revealing that metabolically healthy obesity does not correlate with an increased risk of depression. Instead, the link between obesity and depression seems to hinge on the metabolic profile of the individual.

Moradi et al. (4) identified a statistically significant positive correlation between the prevalence of obesity and the likelihood of experiencing body dissatisfaction among both male and female children and adolescents. However, the association between weight excess and depression reached statistical significance solely in the case of girls (4). The authors hypothesized that the gender-specific correlation observed could be explained by psychosocial factors, particularly weight perception. Their suggestion implied that only those children who perceive themselves as overweight may develop a negative body image, subsequently contributing to the onset of depression (14). Interestingly, this relationship between body dissatisfaction and increasing BMI was less conspicuous in male children, where a higher BMI might be associated with qualities such as strength and athleticism, in contrast to their female counterparts (21). Therefore, while the association between depression and body dissatisfaction, as well as between obesity and a negative body image, appears evident in both genders, the prevalence of depression among obese females is more pronounced (4, 17, 18).

In contrast, to the aforementioned findings, Rao et al.'s (22) meta-analysis of 11 comparative studies involving 69,220 subjects revealed a significant increase in the risk of major depressive disorder (MDD) among children and adolescents with obesity when compared with healthy controls. Notably, this increased risk was observed without any gender-specific differences (17).

A key distinction between this meta-analysis and the previously mentioned studies lies in the criteria for diagnosing depressive disorder. Rao et al. (22) uniquely included studies where the diagnosis of depressive disorder was based on international diagnostic criteria, such as the International Classification of Diseases, eleventh version (ICD-11), and DSM-5 (10, 16). This selective focus on standardized diagnostic criteria limited the generalizability of the findings for clinical practice.

Another noteworthy aspect of Sutaria et al.'s (17) study is the execution of a subgroup meta-analysis specifically focusing on longitudinal studies, encompassing follow-up periods ranging from 1 to 20 years in cohort studies. This analysis unveiled an association between childhood obesity and elevated odds of developing depression in the future when compared to normal-weight children (17). This finding aligns with the understanding that the onset of depression during childhood augments the risk of experiencing subsequent depressive episodes in adult life (23).

### 3.2 Pathogenic mechanism of obesity and depression development

Many studies discuss multiple common pathological mechanisms involved in obesity and depression like chronic systemic inflammation (24), the dopaminergic reward system, vitamin D deficiency (25) and neuroendocrine mechanisms via leptin melanocortinergic—BDNF signaling (9).

The mechanisms behind the association between diet and -both mental and physical- health outcomes are complex, multifaceted and not restricted to any one biological pathway. Among the pathways implicated, the hyperactivation of the hypothalamus–pituitary–adrenal axis, and the consequent excessive glucocorticoids secretion, seems to have an important role both mood disorders and obesity (8, 26). Indeed, it was shown that stress and HPA-axis activation causes overconsumption of Western- style food and subsequent obesity (27). This phenomenon (known as emotional eating and comfort food) acts as attempt to mitigate stress and anxiety (27).

Another important player in this context is the neuronal reward circuitry and the neurotransmitters involved in it. Specifically, a correlation between exposure to high-fat diet (highly prevalent in subjects with obesity) and increase in serotonin and dopamine, acting on mood regulation, has been found (8, 26).

Lastly, inflammation levels seem to have a role in both mood disorders and obesity (28). Interestingly, Miller et al. reported in a cross-sectional study that depressive symptoms promote weight accumulation, that in turn activates an inflammatory response through adipose tissue release of interleukin-6 and leptin-induced upregulation of interleukin-6 release by white blood cells (29).

Indeed, obesity is characterized by chronic low-grade inflammation and depressed subjects present higher levels of inflammatory markers vs. controls according to clinical studies on cytokine-induced depression and comprehensive meta-analyses (30). In the cross-sectional study McLachlan et al. (11) demonstrated greater levels of inflammatory cytokines (C-reactive protein, tumor necrosis factor-alpha, interleukin-6) and adiposity measures such as BMI percentile, trunk/total fat ratio and total fat in depressed adolescents compared to non-depressed counterparts. Additionally, also severity depression showed a significantly positive correlation with BMI percentile and visceral adipose tissue. Thus, among therapeutic opportunities, it's crucial to focus on managing body composition reducing chronic inflammation to potentially improve outcomes (11).

Similarly, major depressive disorder is associated with expressions of genes involved in innate and adaptive immune pathways.

Neuroendocrine substances are also implicated in this intricate etiopathogenesis. Leptin influences mood acting on neuron receptors expressed in the limbic system, strengthening of neurogenesis and cortex neuroplasticity and modulation of hypothalamic-pituitary-adrenal (HPA) axis and immune system. Primary obesity is characterized by leptin resistance which is considered a possible risk phenotype for depression (31, 32). Brain regions that regulate appetite and energy (hypothalamus and pituitary gland) and mood regulation (hippocampus and limbic system) contain highly expressed genes near loci associated with BMI. These findings demonstrate its crucial role in regulating body mass and energy homeostasis, which intersect with those responsible for mood regulation (33). Neuroimmodulation and neuroplasticity vitamin D's functions could imply its role in psychiatric disorders, biologically proved also by the presence of vitamin D receptors in cerebral regions involved in depression (25). Hypothalamic-pituitary-adrenal axis dysregulation and hyperactivation is another mechanism widely reported. Long-term exposure to a high level of stress hormone like cortisol increases appetite, promotes adipogenesis and reduces energy expenditure, but also causes neurological lesions of the limbic areas (hippocampus and amygdala) affected in depression. It is plausible that the hypercortisolism of patients with obesity makes them more susceptible to metabolic complications of obesity and depression (34). These findings impact appetitive and homeostatic regulatory systems promoting obesogenic and depressogenic behaviors. In Figure 1, pathogenic factors involved in childhood obesity and depression are schematized.

## 4 Interaction between mood, emotional state, and feeding behaviors

### 4.1 Regulation of energy intake in humans

Energy balance requires that an organism match caloric intake relatively precisely with caloric expenditure (35, 36). In humans, it has been estimated that an error of only +11 kcal/day results in a one pound weight gain over the course of a year (35).

In order to face obesity and its consequences, both from the physical and from the psychological point of view, it's critical to understand the mechanisms which regulate energy homeostasis and food intake in humans.

Interesting, pioneer studies of animal behavior highlighted the existence of two mechanisms involved in the regulation of food intake: satiation and satiety (37). Satiation is defined as a set of complex processes that inhibit the motivation to eat during an eating event. Generally, the beginning of a meal is due to different stimulations, involving physiological factors (hunger), sensory cues (olfactory, gustatory, visual), and other factors linked to the environment (time of day, mood, the social situation); progressively, food is eaten internal inhibitory influences (sensory, cognitive, gastric, hormonal, neural) develop and bring ingestion to an end (37, 38).

Satiety instead is the inhibitory mechanism occurring at the end of eating and prevents the return of hunger for variable time. In efficient appetite and energy control, hunger, satiation, and satiety occur in succession and balance energy intake to energy needs. Instead, the current epidemic of obesity shows that different things can alter appetite control and energy intake, because of various environmental and psychological influences, as emotional eating (EE), tend to override physiological mechanisms (37). In order to better understand this issue, it's fundamental to briefly review the physical mechanisms involved in energy intake regulation.

Food intake is regulated by activation of peripheral signals, the so called “afferent system” (e.g., in the gastrointestinal tract and adipose tissue), that directly monitor incoming nutrient and nutrient stores, and central systems (e.g., the hypothalamus, the hippocampus, the hindbrain), which receive these signals and modify behavioral and metabolic output to balance energy intake through the “efferent system”. These signals interact with one another, making other signals more or less effective (35, 37).

In Table 2, the main key peptides and neurotransmitters involved are resumed (39).

**Table 2:** Key peptides and neurotransmitters involved in the regulation of energy intake (39).

	Stimulation of production site	Production inhibitors	Effects on the regulation of hunger	Effects on energy expenditure
Afferent system				
Leptin	Post-prandial period	Fasting	Anorexigenic action	Increases
Ghrelin	Fasting/pre-prandial period	Post-prandial period	Orexigenic action	Decreases
Glucagon like-peptide -1 (GLP-1)	Post-prandial period	Fasting	Anorexigenic action	No effects
Insulin	Post-prandial period	Overnight fasting, preprandial period	Anorexigenic action	No effects
CNS system				
Neuropeptide (NPY)	Fasting/Ghrelin	Leptin, glucose	Orexigenic action	Decreases
Agouti-related peptide protein (AgRP)	Fasting/Ghrelin	Leptin	Orexigenic action	Decreases
Melanin-concentrating hormone (MCH)	Flavors of food	Melanocyte-stimulating hormone (MSH)-producing neurons	Orexigenic action	No effects
Alpha-melanocyte-stimulating hormone (a-MSH)	Leptin	AgRP	Anorexigenic action	Increases
Efferent system				
Irisin	Exercise, energy expenditure	Unknown	No direct effect on hunger	Increases
Serotonin/Norepinephrine	Environmental factors: low temperatures, diet, mood	Weight loss	Orexigenic action	Increases
Thyroid hormones	Low temperatures, high caloric diet	Critical illness, caloric restriction, drugs	Appetite variations according to hyper or hypothyroidism	Increases

Interestingly, overweight, or chronic consumption of a high-fat, calorie-rich diet, has been correlated to a change in the sensitivity to these signals and a decreased ability to regulate energy balance and satiety (35, 40). Moreover, obesity seems to affect also cognitive function and sensitivity to the rewarding aspects of food (35). This may further contribute to the persistence of weight gain and overweight status (35, 40).

### 4.2 Emotional eating

Individuals engage in diverse methods to manage their mood, with food consumption being a prevailing approach (41). The interaction between mood and food intake is reciprocal, involving both the emotional well-being of the individual and the selection and amount of food consumed (42).

Emotional eating, characterized by eating in reaction to emotions, especially negative ones like sadness, anger, or boredom, and notably when not physically hungry, is a recognized feeding behavior (43–56). This dietary pattern often involves eating foods that provide pleasure, serving as a means able to alleviate negative emotional states through hedonic satisfaction (47, 48). Notably, EE can begin as early as the age of two (46, 49). While the exact origins of EE remain unclear, heritability estimates suggest a minimal role for genetic factors, suggesting that EE is predominantly a learned behavior, driven mainly by environmental factors (50).

Therefore, mood effect on reward mechanisms influenced by food intake is well-established (26). Notably, in specific emotional states, certain foods are chosen for their impact on the reward-processing areas of the brain (42, 51–53).

Intriguingly, highly appealing foods have been shown to stimulate brain regions tied to reward and pleasure, in a manner akin to the neural activations seen in drug addiction (54). This suggests the existence of a specialized neural mechanism for food addiction, which plays a significant role in prompting overeating and contributing to the development of obesity (42, 55–59).

Two parallel signaling systems, interoceptive and exteroceptive, ensure normal eating behavior by finely regulating energy intake and the emotional-motivational processes underlying feeding (53, 60). Endocrine and metabolic signals regulate energy consumption and expenditure in response to the body's requirements, through their interaction with the hypothalamic and brainstem nuclei (53). This system is interconnected with a different neural system that oversees the hedonic pleasure derived from food consumption, eliciting emotional responses via the brain's reward system (53, 60, 61). This includes regions like the substantia nigra and ventral tegmental area, along with several cerebral areas such as the amygdala and the orbitofrontal cortex (53, 60, 61). Together, these systems assimilate signals pertaining to the nutritional and sensory state of food, consequently affecting its rewarding qualities, the appetite, and eating behaviors (62, 63). Under typical conditions, the homeostatic and hedonic pathways interact and mutually influence each other. However, in scenarios involving stress or disorders like obesity or depression, this delicate equilibrium can be disrupted (53, 64, 65).

Alterations in dietary habits often serve as a key indicator of mood disorders (42). As stated before, there is a notable and significant link between obesity and depression, a relationship well-documented through extensive research in both human and animal models (42, 66–69).

Preferring palatable foods seems to alleviate stress and anxiety symptoms (70–72). Such dietary patterns are observed in both humans and animals (73–76), suggesting the involvement of a common neurobiological pathway in the decision-making process regarding food choices and eating habits under stress.

Consuming highly palatable, calorie-dense “comfort foods” appears to offer a transient amelioration of depressive symptoms (42). Though, chronic adherence to a high-fat diet is associated with the induction of negative affective states, increased stress sensitivity, and alterations in the basal levels of corticosterone (42). This scenario culminates in a recurrent pattern of excessive food intake, progressive weight gain, and deteriorating mood states, thereby perpetuating a cycle of overeating and psychological distress.

Numerous systematic reviews and meta-analyses have highlighted substantial evidence linking specific dietary patterns, overall diet quality, and various food groups, including macro- and micronutrients, with the onset of depression and the severity of its symptoms (3, 77, 78).

Furthermore, food, mood, and stress are intricately linked (42, 68, 79). Stressors, whether acute or chronic, significantly affect eating behaviors, leading to either an increase or decrease in both the quantity and quality of food consumed (68, 79, 80).

## 5 Dietary habits and the risk of depression

The nutritional quality of children's diet has markedly declined in recent decades. This low-quality diet may adversely affect children's health, increasing the risk of dental issues, childhood obesity and related complications, subpar academic performance, and diminished self-esteem. This deterioration, coupled with a seemingly parallel rise in the prevalence of adolescent depression, has spurred increased interest in evaluating the potential dietary role in the development or exacerbation of depressive symptoms (81–85). Considering the developmental processes of brain during childhood, the influence of diet on mental health may arguably be more significant during this period than in subsequent stages of life (86). It is crucial to consider as confounding variables various influencing factors conditioning socio-economic status as these can impact diet. On the whole, researches focusing on adults and their diet's relationship with mental health have suggested a multifaceted and potentially bidirectional connection (86).

In epidemiological studies examining correlations among adults, a Westernized diet has been associated with a heightened likelihood of mental disorders and psychiatric unease. Conversely, it has been demonstrated that a healthy diet is related to enhanced mental well-being (81, 87, 88). Similarly, a study conducted by Jacka et al. (89) has revealed that adolescents adhering to a healthy diet exhibit a lower likelihood of reporting symptomatic depression. On the contrary, adolescents who consumed a higher amount of processed foods have a higher risk of experiencing depression. Furthermore, a year later, Jacka et al. has demonstrated that betterment of eating habits is correlated with improvement of mental health (89). Converging results have been found out by a recent meta-analysis conducted by Orlando et al. this research revealed a noteworthy association between adopting a healthy eating pattern and fewer depressive symptoms among children and adolescents (90).

Over the years, various studies have examined the influence of diet on mood disorders, and a correlation has been observed between depression and sweetened beverages by Zahedi (91) and Hoare et al. (92), take-out and fast food consumption by Castillo (93) and Zahedi et al. (91), daily salty snack intake by Zahedi et al. (91). Therefore, findings from these studies indicate that foods with elevated levels of starch, fat, and salt exhibit a more pronounced association with depressive symptoms compared to sweet foods.

Recent observational research has underscored that individuals grappling with severe mental illness display markedly heightened levels of dietary inflammation when compared to the broader population. More specifically, children and adolescents grappling with major depressive disorders exhibit heightened levels of pro-inflammatory cytokines and this escalation in pro-inflammatory markers served as a predictive factor for depression symptoms in this age group (30, 94). Furthermore, unhealthy eating behaviors contribute to the worsening of depression symptoms by fostering increased inflammation. The latter derives from an increased intake of pro-inflammatory foods, including refined carbohydrates and trans fats, coupled with diminished consumption of anti-inflammatory nutrients sourced from whole foods and plants. Additionally, meta-analyses of longitudinal studies has demonstrated that individuals adhering to a more inflammatory dietary pattern face a heightened risk of developing depression over an extended period (8, 95).

In Dehghan et al.'s research (96), a substantial negative association has been observed between dietary antioxidant index and depression in adolescent girls. This emphasizes the significance of maintaining a healthy and anti-inflammatory nutrition for adolescents' mental well-being (96). As demonstrated by a recent study conducted by Sureda et al. (97), adolescents who more strictly adhere to the Mediterranean diet exhibit lower levels of C reactive protein. All these pieces of evidence support how enhanced compliance with the Mediterranean diet has the potential to mitigate the impact of inflammation induced by stress and lower the likelihood of future mental health issues (98). A healthy diet encompasses various nutritional components, with certain elements standing out for their anti-inflammatory properties.

### 5.1 Foods and nutrients associated with depression

Phytochemicals like polyphenols found in blueberries, and cocoa exhibit strong anti-inflammatory effects (99). Eicosapentaenoic acid and docosahexaenoic acid, omega-3 fatty acids abundant in marine products like salmon, have anti-inflammatory properties. These fatty acids also demonstrate a capacity to delay cytokine-induced depression (100, 101). Moreover, results from studies on animals have indicated that omega-3 fatty acids may mitigate inflammation-induced decreases in neurogenesis to a degree similar to that of antidepressants (8). Oxidative stress is implicated in cellular damage associated with depression. The meta-analysis of 115 articles conducted by Liu et al. (102) has shown elevated oxidative stress markers and reduced antioxidant markers in individuals with depression compared to healthy controls (102). Antidepressant treatment is linked to a reduction in oxidative stress markers, suggesting a causal relationship. Moreover, diet plays a pivotal role, influencing oxidative stress by either depleting or enhancing dietary compounds with antioxidant properties (102). Studies on animals, such as the research conducted on aged mice by Morrison et al. (103), have suggested that high-fat Western-style diets can raise indicators of oxidative stress both in brain and periphery. Given the reported elevated levels of oxidative stress in individuals with mental disorders, improving dietary quality emerged as a potential strategy to restore oxidative balance (8). Vitamins C and E as direct free radical scavengers and selenium, zinc and cysteine as cofactors for antioxidant system combat oxidative stress (8). Furthermore, according to Zhang et al. (104), also polyphenols enhance antioxidant defenses nuclear factor erythroid, nuclear factor k B and mitogen-activated protein (MAP) kinase pathway signaling (104). Polyphenols have demonstrated the ability to influence the gut microbiota and function as anti-inflammatory agents (105).

Some of above-mentioned micronutrients have a potential antidepressant action, independent from the antioxidant effect. Vitamin C, beyond antioxidative properties and consequent neuroprotective action, serves as a neuromodulator within the brain, influencing neurotransmission mediated by both dopamine and glutamate and exert an impact on 5- Serotonin 1A (HT1A) receptor (106–109). Zinc regulates N-methyl-D-aspartate (NMDA) receptor function and boosts neuroplasticity and neurogenesis. This micronutrient impacts serotonin receptors and regulates immune responses (107). Vitamin D exhibits various potential mechanisms with antidepressant effects: it modulates innate and adaptive immune response through regulation of inflammation and production of antimicrobial substances, it influences hypothalamic-pituitary-adrenal axis, and it regulates serotonin and dopamine synthesis (110–112). A recent umbrella review published by Xu et al. (3), has affirmed an inverse link between depression risk and intake of fruits and vegetables, particularly noticeable in high vs. low consumption meta-analysis, albeit with evidence of modest quality. A heightened presence of saturated fats in red and processed meat has been associated with reduced brain-derived neurotrophic factor, irregular neuroplasticity, and compromised cognitive function—all implicated in depression's pathogenesis.

Conversely, fish and nuts, abundant in n-3 polyunsaturated fatty acids, exhibited potential benefits in preventing depression through anti-inflammatory processes, neuro-endocrine modulation, and neurotransmitter activation (3). The ANIVA (Antropometria y Nutricion Infantil de Valencia) study, a descriptive cross-sectional study conducted by Rubio-Lopez et al. (81), has shown a reduced intake of carbohydrates in children displaying symptoms of depression than those without such symptoms.

Carbohydrates exert a profound influence on nervous system, mood and behavior: their ingestion induces to supply glucose and energy, and conditions neurotransmitter synthesis. The consumption of a high amount of carbohydrates induces the release of insulin, facilitating the entry of blood sugar into cells for energy while simultaneously promoting the entry of tryptophan into the brain (81).

As shown by Teesson et al. (113) and Chou et al. (114), alcohol consumption by adults is linked to depression. The association between alcohol and depression has been reported, with past cross-sectional studies suggesting a higher susceptibility to depression in individuals facing alcohol-related issues. However, it is crucial to note that those with depression may drink alcohol as a means for alleviating distress, potentially leading to an overestimation of alcohol's impact on depression risk.

In psychiatry, the exploration of tryptophan availability and metabolism has predominantly focused on its conversion into serotonin, a pivotal target for most antidepressants. Nevertheless, the primary physiological route for tryptophan entails the kynurenine pathway, giving rise to both neurotoxic quinolinic acid and neuroprotective kynurenic acid (115). Exploring tryptophan supplementation as a strategy in managing depression, studies have yielded diverse results. Within the brain, tryptophan influences neurotransmitter levels as the precursor to brain serotonin. Individuals with diminished brain serotonin levels are deemed susceptible to depression (8, 81). However, in instances of heightened tryptophan metabolism through the kynurenine pathway (e.g., induced by stress or immune activation), there could be an augmented generation of the neurotoxic quinolinic acid. As shown by O' Connor et al. (116) giving L-kynurenine to mice with no prior exposure results in dose-dependent induction of behavior resembling depression. An essential cofactor in the metabolism of tryptophan is vitamin B6 that acts facilitating its conversion into the neurotransmitter serotonin.

Vitamins from the B-complex play a central role in energy metabolism, mitochondrial function, and the production of neurotransmitters (107, 117). The cross-sectional analysis conducted by Murakami et al. has demonstrated a negative correlation between riboflavin level and depression in females aged 12–15 years, but no association with B12 levels in both sexes (118). In another cross-sectional study conducted by Herbison et al. (119) females and males aged 17 years characterized by a low intake of vitamin B6 and folate had higher internalizing behavior scores measured by Youth Self Report. Although mechanisms behind are unknown, also caffeine consumption seems associated with a reduced risk of depression. Several theoretical biological rationales exist, according a plausible theory coffee, with its abundant caffeine content, can stimulate the central nervous system and augment dopaminergic neurotransmission (3). As demonstrated by studies conducted with animal models, high fat or high calories diet can modify gut microbiome increasing Firmicutes/Bacteroidetes ratio (120) and Clostridiales, Ruminococcaceae, and Bacteroidales (121) contributing to behavioral changes similar to symptoms of depression and anxiety. As shown by Hiel et al. (122), a diet rich in inulin-type fructans, a type of fermentable dietary fiber, increases Bifidobacterium spp., enhancing satiety and intrapersonal competence without affecting mood or perceived stress (122). Liu et al. (123) through a random-effects meta-analysis of 34 controlled clinical trials evaluating the effects of probiotics and prebiotic on depression and anxiety has shown that addition of live microorganisms, either Lactobacillus spp. alone or in conjunction with Bifidobacterium spp., has the potential to positively impact both depression and anxiety. Contrariwise the use of prebiotic has shown no significant difference in symptoms compared to control, but the sample was limited and composed especially by non-clinical participants. Nevertheless, according to the major literature, also prebiotic supplementation reverses stress-induced microbiota changes preserving beneficial Bifidobacterium spp. or Lactobacillus spp. and mitigates depressive behaviors in mice. Fermented foods, incorporating functional microorganisms, prebiotics, and biogenics, constitute a food category with the potential to influence gut-brain communication (123, 124). Exact pathways which connect the gut microbiota to diet-brain are still under study, but various mechanisms have been suggested, such as through immune-immunomodulatory action of microbial metabolites as short-chain fatty acids which derives from dietary fiber, neuronal and endocrine pathways through nervus vagus and microbioma direct-indirect control of tryptophan metabolism pathway or of other neurotransmitters such as *γ*-Aminobutyric acid (GABA) and the hypothalamus–pituitary–adrenal axis (8). Among studies suggesting involvement of hypothalamic-pituitary-adrenal axis, the one conducted by Barbadoro et al. (125) has highlighted improvement in cortisol levels in both healthy and depressed adult through administration of omega-3 fatty acid. Moreover, the study conducted by Tsang et al. (126) has demonstrated similar result through the administration of polyphenol-rich foods in adults recruited from a health and social care setting. The involvement of hypothalamic-pituitary-adrenal axis seems implicated in the pathophysiology of neuropsychiatric disorders: more than 60% of individuals with depression display heightened cortisol production or disruptions in this axis (8).

Results from animal studies have indicated that an elevated sugar diet during the puberal transition fosters increased depressive tendencies in adulthood by activating the hypothalamic-pituitary-adrenal axis and elevating glucocorticoids. Considering that teenagers constitute the major consumers of sugar-sweetened beverages, an overabundance of added sugar during this developmental phase could contribute to the enduring dysregulation of stress response and predispose to depressive symptoms (3, 127). Also, mitochondrial dysfunction is linked to depression and extensive preclinical data indicated that an inadequate diet might play a role in causing mitochondrial dysfunction. As illustrated by Kuipers et al. (128), Sihali-Beloui et al. (129) and Woodman et al. (130), respectively a high fat diet a hypercaloric diet rich in carbohydrate and a diet high in salt are linked to irregular mitochondrial biogenesis, a phenomenon also correlated with heightened free radical generation and inflammation, conditions associated with depressive symptoms.

In Table 3, a list of foods and nutrients associated with higher or lower risk to develop depression are resumed.

**Table 3:** Foods and nutrients associated with higher or lower risk to develop depression.

Increased risk	Lower risk
Higher consumption of processed foods (89)	Adequate consumption of polyphenols (99, 104)
Higher consumption of sweetened beverages (91, 92)	Adequate consumption of eicosapentaenoic acid (100, 101)
Higher consumption of take-out and fast food (91, 93)	Adequate consumption of docosahexaenoic acid (100, 101)
Higher consumption of salty snack (91, 130)	Adequate consumption of vitamin C (8)
Higher consumption of refined carbohydrates (8, 95)	Adequate consumption of vitamin E (8)
Higher consumption of trans fat (8, 95)	Adequate consumption of vitamin D (110–112)
Higher consumption of fat (120, 128)	Adequate consumption of vitamin B complex (118, 119)
Higher consumption of alcohol (113, 114)	Adequate consumption of cysteine (8)
Higher consumption of tryptophan/kynurenine elements pathway (115, 116)	Adequate consumption of selenium (8)
Higher consumption of calorie-dense diet (121)	Adequate consumption of tryptophan (115)
Reduced intake of carbohydrates (81)	Adequate consumption of zinc (8, 107)
Higher consumption of hypercaloric diet rich in carbohydrate (129)	Higher consumption of fruit and vegetables (3)
	Higher consumption of fish and nuts (3)
	Adequate consumption of caffeine (3)

### 5.2 Gut microbiota and the risk of depression

As demonstrated by studies conducted with animal models, high fat or high calories diet can modify gut microbiome increasing Firmicutes/Bacteroidetes ratio (120) and Clostridiales, Ruminococcaceae, and Bacteroidales (121) contributing to behavioral changes similar to symptoms of depression and anxiety. As shown by Hiel et al. (122), a diet rich in inulin-type fructans, a type of fermentable dietary fiber, increases Bifidobacterium spp., enhancing satiety and intrapersonal competence without affecting mood or perceived stress (122). Liu et al. (123) through a random-effects meta-analysis of 34 controlled clinical trials evaluating the effects of probiotics and prebiotic on depression and anxiety has shown that addition of live microorganisms, either Lactobacillus spp. alone or in conjunction with Bifidobacterium spp., has the potential to positively impact both depression and anxiety. Contrariwise the use of prebiotic has shown no significant difference in symptoms compared to control, but the sample was limited and composed especially by non-clinical participants. Nevertheless, according to the major literature, also prebiotic supplementation reverses stress-induced microbiota changes preserving beneficial Bifidobacterium spp. or Lactobacillus spp. and mitigates depressive behaviors in mice. Fermented foods, incorporating functional microorganisms, prebiotics, and biogenics, constitute a food category with the potential to influence gut-brain communication (123, 124). Exact pathways which connect the gut microbiota to diet-brain are still under study, but various mechanisms have been suggested, such as through immune-immunomodulatory action of microbial metabolites as short-chain fatty acids which derives from dietary fiber, neuronal and endocrine pathways through nervus vagus and microbioma direct-indirect control of tryptophan metabolism pathway mentioned above or of other neurotransmitters such as GABA and the hypothalamus–pituitary–adrenal axis (8).

Exploring the immune modulation along the brain-gut-microbiota axis, extensive research has delved into variations in inflammatory cytokines to probe the immune dynamics in depression. Nonetheless, the intricate immune signaling network underlying these inflammatory fluctuations, encompassing innate and adaptive immune modulation within the gut, brain, and systemic circulation, has surfaced as an integral component of the communication framework among the microbiota, gut, and brain in depression (131, 132).

In 2015 Erny et al. demonstrated the importance of the host microbiota in microglial maintenance, with germ-free mice showing widespread microglial abnormalities characterized by an immature phenotype and defects in innate immunity. Reintroducing a diverse microbiota partially restored normal microglial characteristics. Glial cells engage with neurons, impacting brain well-being and conditions like depression (132, 133).

The gut microbiota can influence glial functions via neural and chemical signaling pathways, modulating microglial activation and playing a role in triggering neuroinflammatory processes in depression (131).

Moreover, research indicates that Th17 and Treg cells, influenced by the gut microbiota, play a crucial role in depression, impacting neuroinflammation and immune balance in the brain and gut (134).

Imbalances in (Thelper) Th17/T regulator (Treg) ratios are linked to depression, affecting the brain-gut-microbiota axis. Specific gut microbiota (e.g., Clostridia, Bacteroides fragilis, Lactobacillus reuteri, and Bifidobacterium) and their metabolites also shape Th17/Treg activity, influencing susceptibility to stress and antidepressant effects (135). Further studies are needed to understand this interplay fully.

Research suggests that commensal microbes in the mammalian gastrointestinal (GI) tract significantly influence intestinal tryptophan availability, impacting tryptophan metabolism. While most ingested tryptophan is absorbed in the small intestine, some reaches the large intestine, where commensal microbes degrade it into tryptamine, a serotonin-related compound (136). Germ-free mice lacking microorganisms show reduced tryptamine levels but increased tryptophan in blood, suggesting microbiota involvement in tryptophan metabolism modulation (137). Additionally, gut microbiota metabolizes tryptophan into indole derivatives and affect serotonin levels in various ways, including promoting serotonin biosynthesis in colonic cells and directly producing serotonin. Microbial modulation of the kynurenine pathway, influenced by inflammatory mediators and microbial metabolites like short-chain fatty acids, further highlights the intricate relationship between the gut microbiota and tryptophan metabolism, with implications for immune function and neuroactive compound production (56, 136).

Particular strains of Lactobacillus and Bifidobacterium produce gamma-aminobutyric acid, a primary inhibitory neurotransmitter in the central nervous system. According to literature, disrupted or altered gamma-aminobutyric acid signaling pathways are associated with anxiety disorders and depression (138, 139). In individuals with unipolar depression, GABA levels in cerebrospinal fluid and plasma are reduced compared to those in control subjects (139). In 2002 Sanacora and co-authors (140) have utilized *in vivo* proton magnetic resonance spectroscopy to demonstrate decreased GABA levels in the occipital cortex of depressed patients, with a elevation observed in patients undergoing treatment with selective serotonine reuptake inhibitors (139, 140).

Depression correlates with the signaling of the vagus nerve, which plays a role in regulating inflammation and is influenced by neuroactive substances. The vagus nerve establishes a vital connection between the brain, the microbiota in the gut, and the immune system (132). In 2011 Bravo et al. discovered that oral administration of Lactobacillus rhamnosus mitigated stress-induced depressive behaviors in mice when compared to the control group. Furthermore, they found that vagotomy hindered the effects of Lactobacillus rhamnosus on neurochemical processes and on behavioral effects (132, 141). As shown by Wang et al. in 2020 (142), consumption of microbes related to depression such as Lactobacillus intestinalis and Lactobacillus reuteri resulted in depression-like characteristics, elevated levels of interleukin (IL)-6 in the bloodstream and decreased synaptic protein expression in the prefrontal cortex of antibiotic-treated mice. Remarkably, selective dorsal vagotomy prevented the onset of depression-like behaviors, rise in plasmatic IL-6 levels, and decline in synaptic proteins in the prefrontal cortex following oral introduction of Lactobacillus intestinalis and Lactobacillus reuteri (132, 142). These studies acknowledge the vagus as a crucial regulatory communication pathway linking gut-exposed bacteria to the brain.

Among studies suggesting involvement of hypothalamic-pituitary-adrenal axis, the one conducted by Barbadoro et al. (125) has highlighted improvement in cortisol levels in both healthy and depressed adult through administration of omega-3 fatty acid. Moreover, the study conducted by Tsang et al. (126) has demonstrated similar result through the administration of polyphenol-rich foods in adults recruited from a health and social care setting. The involvement of hypothalamic-pituitary-adrenal axis seems implicated in the pathophysiology of neuropsychiatric disorders: more than 60% of individuals with depression display heightened cortisol production or disruptions in this axis (8).

## 6 Benefit of diet interventions on obesity and depression

It's widely known that diet is the core intervention in the treatment of obesity, both in adulthood and in the pediatric field (143). Interestingly, growing evidence supports the potential use of dietary interventions also as an adjunctive treatment for depression and mood disorders, either associated or not with obese phenotype (144). Indeed, accumulating evidence indicates that obesity and mood disorders are intrinsically linked (26) and the two conditions share clinical, neurobiological, genetic and environmental factors (26).

The emerging area of research working on the potential use of nutritional intervention as adjunctive treatment for depression and mood disorders is known as “Nutritional Psychiatry” (144–146). The dietary interventions studied in this context include nutrient interventions, food interventions and/or whole diet interventions (e.g., Mediterranean or Ketogenic diet) (8).

Unfortunately, there is still insufficient evidence on nutrient-intervention in the treatment of mood disorders as the results of the meta-analyses are still conflicting (144, 145, 147) and evidences lack in the pediatric field. Table 4, reassumes the nutrients studied and the mechanisms of action proposed (144). However, among the supplements studied, omega-3 polyunsaturated fatty acids (n-3 PUFAs) have the greatest evidence for use in the treatment of mood disorder, especially eicosapentaenoic acid (EPA) (148). The beneficial role of n-3 PUFAs has also been shown in subjects with obesity, with and without mental disorders (158, 159), but studies on children are still lacking.

**Table 4:** Nutritional supplements tested in the treatment of depression/mood disorders—adapted from (144).

Nutrient	Mechanism of action	Sources	Effects	Reference
Omega-3 polyunsaturated fatty acids (EPA and DHA)	Anti-inflammatory and antioxidant actions	Fish and fish oil	Beneficial effects in adding n–3 PUFAs to antidepressant therapy, but still inadequate evidence for using n–3 PUFAs as monotherapy for depression/mood disorders	(148, 149)
Zinc	Anti-inflammatory and antioxidant actions	Seafood, beans, nuts, red meat, whole grains and dairy products	Only empirical evidence supports an inverse association between zinc supplementation and depressive symptoms	(3, 150)
Vitamin D	Gene expression regulation, promotes anti-inflammatory and antioxidant actions; immune modulation; involved in production of neurotransmitters	Fish (e.g. salmon, tuna and mackerel), egg yolk, liver and cheese	Vitamin D supplementation favorably impacted depression ratings in major depression with a moderate effect size	(151, 152)
Vitamin B9/ Folate	Not clear—Low folate status associated with depression and decrease the efficacy of antidepressant treatment	Vegetables (dark green leafy, spinach etc) and liver	folate supplementation seems to improve the efficacy of traditional antidepressant medications, but further research needed	(153, 154)
S-Adenosylmethionine (SAMe)	Control in the DNA methylation and synthesis of neurotransmitters	Synthesized from adenosine triphosphate and the alpha-amino acid methionine	AMe adjuvant therapy correlated to increase proportions of antidepressant responders, or improve the speed of response to antidepressants	(155, 156)
N-acetylcysteine	Anti-inflammatory and antioxidant actions	Derivative of the amino acid cysteine	No specific improvements in symptoms identified when adding N-acetylcysteine to anti-depressant treatments	(147, 157)

EPA, eicosapentaenoic acid; DHA, docosahexaenoic acid; PUFAs, polyunsaturated fatty acids.

The Mediterranean diet (MD), detected in the 1950s and 1960s among Mediterranean populations, emphasizes consumption of plant-based foods, moderate fish, and olive oil as the main fat source. The diet is based on moderate intake of white meat, low-fat dairy, eggs, and low red wine for adults, whereas the consumption of red and processed meats, and sweets is limited. (MD) values seasonality, biodiversity, and consumption of traditional local foods (77, 160, 161). On the contrary, the prevalent Western diet in developed countries leans towards convenience and highly processed foods. It involves high intake of processed and red meat, fried foods, sweets, high-fat dairy, and refined grains, with low consumption of plant-based foods such as whole grains, legumes, vegetables, and fruits.

Among the whole diet interventions studies, different meta-analyses of observational studies evidenced the association between the (MD) and decreased risk of psychiatric disorders (144, 162–164). Specifically, the protective effect of MD against depression was recently confirmed by Lasse et al. and, previously, by Psaltopoulou et al. (144, 163). Recently, Salari-Moghaddam et al. performed a cross-sectional study on 3,176 subjects and demonstrated that high adherence to a regime called Mediterranean-Dietary Approach to Stop Hypertension (DASH) Diet Intervention for Neurodegenerative Delay (MIND), also decreases the risk of developing depression (165). This dietary intervention was created mixing the MD and the DASH diet (165). Interestingly, Jacka et al. performed a randomized controlled trial, aiming to investigate the efficacy of a dietary improvement program for the treatment of major depressive episodes (166). The randomized controlled trial was called “Supporting the Modification of Lifestyle In Lowered Emotional States” (SMILES) and evaluated the effect of a dietary improvement program based on a modified MD (“ModiMedDiet”) in reducing the severity of depressive symptoms in patients with major depression (166). The ModiMedDiet is similar to the MD, but encourages a moderate consumption of red meat and dairy (166). At the end of the 12 weeks of intervention, the subjects that followed the ModiMedDiet had a lower score for depressive symptoms and a higher frequency of remission (166). However, further studies are needed to confirm the benefits of these dietary regimens for depression and other mood disorders, both in adults and in adolescents and children.

Another dietary plan analyzed in the treatment of neuropsychiatric diseases is the Ketogenic diet (KD) (144, 167, 168). KD, used to treat drug-refractory pediatric epilepsy for over 100 years (169) and known to work also on obesity (170), is becoming increasingly popular for the treatment of other neurological conditions, including mental illnesses (167, 168). This diet consists in a reduction of carbohydrate intake (usually less than 20 g a day), in order to stimulate the production of ketone bodies, used as source of energy by the brain (144). Different promising studies were performed evaluating KD in subjects affected by bipolar disorder (171–173), but works in the children or adolescents affected by depression or other mood disorders are missing.

In sum, the results of the studies cited show the need for more clinical trials to assess the effect of nutritional interventions in the treatment of patients (both adults and children) with mood disorders, either normal weight or obese.

## 7 Conclusions

It is widely recognized that there is a link between mood and food choices, with certain foods selected for their impact on the brain's reward centers. This behavior parallels the one observed in substance addiction, suggesting a specific neural mechanism for food addiction that contributes to overeating and obesity. It is important to note the significant correlation between obesity and depression, indicating a shared biological pathway influencing these conditions. In addition, stress substantially affects eating behaviors, often leading to increased consumption of pleasurable and rewarding foods. This can trigger a cycle of overeating, weight gain, and psychological distress, exacerbating mood disorders and obesity. In addition, consumption of certain types of foods, especially “comfort foods” high in fat and calories, may provide temporary relief from symptoms of depression, but can lead to long-term obesity and further mental health problems.

Understanding these complex interactions is critical to developing preventive strategies focusing on dietary, emotional, and environmental factors, thereby reducing the risk of obesity and mood disorders.

This review suggests that, due to the current paucity of studies in pediatric obesity and depression, for the moment there is not sufficient evidence to drive clear conclusions about the possibility to carry out some therapeutical or preventive strategies in children and adolescence, but only in adults. Moreover, it's worth to underline that in children a specific and rigid dietary plan, as the KD, may need constant clinical evaluations of its possible side effects, with a precise evaluation of growth and nutritional status, in order to avoid possible malnutrition and ensure a linear growth (174). In addition, adherence to this plan may be difficult in the long term, but short nutritional intervention may be anyway sufficient to obtain health improvements (175).

Despite these challenges, dietary interventions including KD represents a possible preventive and therapeutic option for pediatric patients, thus, further research is needed to better understand the potential side effects of this nutritional approach and to find strategies to increase the adherence to the plan.

In conclusion, this analysis highlights the complex relationship between dietary habits, emotional states, and dietary patterns. Therefore, in order to manage both depression and obesity, a holistic and personalized approach, is often necessary and the potential benefits of nutritional interventions should also be considered. Healthcare professionals, including mental health specialists, nutritionists and primary care providers, can provide support for a combination of psychotherapy, medication, lifestyle changes, and other healthcare issues. Integrated care that addresses both mental and physical health is crucial for effective management and long-term well-being.
